# Mitochondrial dysfunction is associated with the severity of liver fibrosis in patients after the Fontan operation

**DOI:** 10.1111/jcmm.18035

**Published:** 2023-11-15

**Authors:** Saviga Sethasathien, Krit Leemasawat, Suchaya Silvilairat, Rekwan Sittiwangkul, Krit Makonkawkeyoon, Apinya Leerapun, Sarawut Kongkarnka, Nakarin Inmutto, Supanai Suksai, Nattayaporn Apaijai, Siriporn C. Chattipakorn, Nipon Chattipakorn

**Affiliations:** ^1^ Division of Pediatric Cardiology, Department of Pediatrics, Faculty of Medicine Chiang Mai University Chiang Mai Thailand; ^2^ Division of Cardiovascular Diseases, Department of Medicine, Faculty of Medicine Chiang Mai University Chiang Mai Thailand; ^3^ Division of Gastroenterology Diseases, Department of Medicine, Faculty of Medicine Chiang Mai University Chiang Mai Thailand; ^4^ Department of Pathology, Faculty of Medicine Chiang Mai University Chiang Mai Thailand; ^5^ Department of Radiology, Faculty of Medicine Chiang Mai University Chiang Mai Thailand; ^6^ Cardiac Electrophysiology Research and Training Center, Faculty of Medicine Chiang Mai University Chiang Mai Thailand; ^7^ Center of Excellence in Cardiac Electrophysiology Research Chiang Mai University Chiang Mai Thailand; ^8^ Cardiac Electrophysiology Unit, Department of Physiology, Faculty of Medicine Chiang Mai University Chiang Mai Thailand; ^9^ Department of Oral Biology and Diagnostic Sciences, Faculty of Dentistry Chiang Mai University Chiang Mai Thailand

**Keywords:** Fontan operation, liver biopsy, liver fibrosis, mitochondrial function

## Abstract

The gold standard for determining the severity of liver disease in Fontan patients is now liver biopsy. Since it is an invasive procedure, this study determined the possibility of applying mitochondrial function from isolated peripheral blood mononuclear cells (PBMCs) as a non‐invasive indicator of liver fibrosis. Fontan patients (*n* = 37) without known liver disease were analysed cross‐sectionally. Patients were classified according to their histology using the METAVIR score as follows; F0/F1—no/mild fibrosis; F2—moderate fibrosis; and F3/F4—cirrhosis. Peripheral blood mononuclear cells were assessed for mitochondrial activity and apoptosis. This study did not find any significant differences in cardiac function among the groups according to liver histology. Interestingly, our findings indicated a significant decrease in maximal respiration and spare respiratory capacity, in both the moderate (F2) and cirrhosis (F3/F4) groups compared with the group without significant fibrosis (F0/F1). Moreover, the cirrhosis group exhibited higher levels of apoptosis and lower levels of live cells, compared with both the moderate and no significant fibrosis groups. In conclusion, the degree of liver fibrosis in Fontan patients is strongly correlated with mitochondrial dysfunction in PBMCs. Mitochondrial function and apoptosis could potentially serve as novel markers for tracking the progression of liver fibrosis in these patients.

## 1 INTRODUCTION

The Fontan operation (or total cavopulmonary connection) is a definitive palliative surgery for single ventricular heart physiology. This surgery was designed to directly connect the total systemic venous vessels with the pulmonary artery without right ventricular pumping ability. Currently, many patients worldwide who have undergone the Fontan operation during childhood can grow into adulthood.^1^ Unfortunately, these patients experience several adverse consequences in multiple organs including central venous hypertension and low cardiac output state, all of which are the hallmark hemodynamic post‐Fontan operation,^2^, ^3^ where high central venous pressure (CVP) is transmitted reverse to the hepatic sinusoid. As a result, chronic liver injury repeatedly occurs. Thus, all Fontan patients develop chronic heart failure post‐Fontan operation. Fontan‐associated liver disease (FALD) is currently one of the most highly recognized challenges for diagnosis and management. There are a variety of severities ranging from congestive hepatopathy, liver fibrosis and cirrhosis to nodular regenerative hyperplasia and hepatocellular carcinoma (HCC).^4^ The gold standard method for identifying FALD is a routine liver biopsy since most patients show no symptoms. Due to the use of oral anticoagulant medications, most patients have a substantial risk of bleeding during the invasive liver biopsy. Therefore, many modalities have been developed to evaluate FALD as a substitute for liver biopsy, including blood tests, echocardiography, upper abdominal ultrasound, ultrasound liver elastography and cardiac catheterization. Unfortunately, when compared to liver biopsy, these modalities were much less accurate. Mitochondria are the organelles that play numerous vital roles for cells such as synthesis of reactive oxygen species (ROS), production of adenosine triphosphate (ATP) and control of cell signalling.^5^, ^6^ When stress occurs, the mitochondrial permeability transition pore (mPTP) is opened due to ROS overload leading to mitochondrial dysfunction and the loss of membrane potential. Finally, the failure of ATP develops and progresses to cell death.^7^, ^8^, ^9^, ^10^, ^11^ Furthermore, mitochondria‐initiated cell death has been shown as the main mechanism contributing to heart failure.^12^ In one study, elevated mitochondrial respiration in peripheral blood mononuclear cells (PBMCs) was correlated with single ventricular heart physiology post‐Fontan operation in patients who developed heart failure, when compared to those without heart failure.^13^ However, there are currently no published studies on the association between mitochondrial function and the severity of liver fibrosis in post‐Fontan patients. Therefore, this study aimed to investigate mitochondrial function for detecting the early stage of FALD based on the METAVIR scoring system by liver biopsy in Fontan patients. We hypothesized that mitochondrial dysfunction in PBMCs is associated with the staging of liver biopsy in Fontan patients.

## 2 MATERIALS AND METHODS

### 2.1 Study population

**FIGURE 1:**
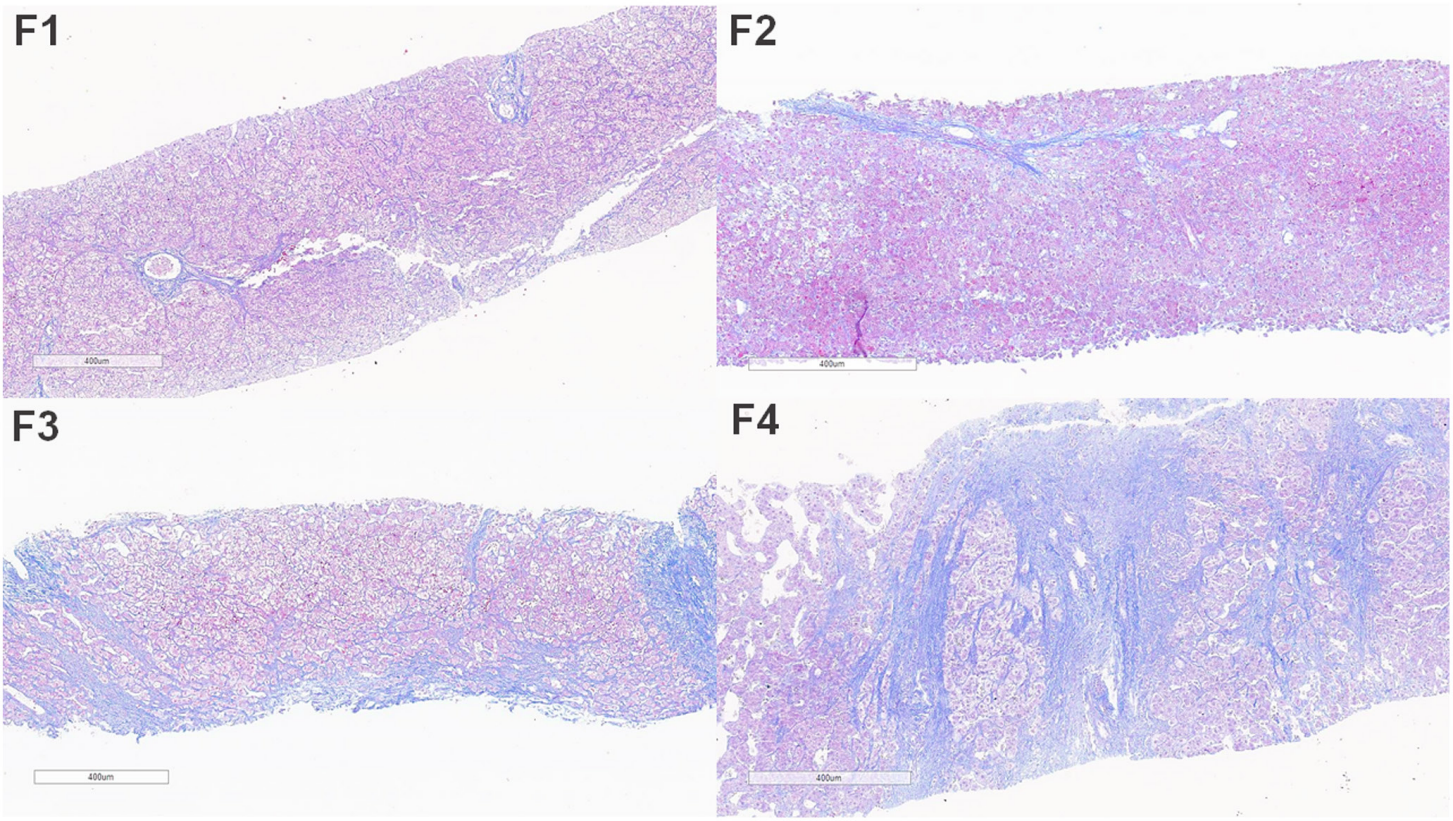
Fibrosis staging of FALD according to METAVIR scoring system. F1, portal fibrous expansion; F2, portal fibrosis with few septa (bridging fibrosis); F3, bridging fibrosis and distorted architecture; F4, probable or definite cirrhosis with nodule formation (mallory trichrome stain). FALD, Fontan‐associated liver disease.

A cross‐sectional study included 37 Fontan patients with the liver biopsy along with liver ultrasound and elastography, computerized tomography (CT) scan of the liver, cardiac catheterization and blood tests at the Chiang Mai University Hospital (Graphical Abstract). Patients with underlying liver disease were excluded. The patients individually consented to all procedures being performed in the same week or within a range of 1 month. This study was approved by the Institutional Review Board of Chiang Mai University Medical Center (Approval no. PED‐2563‐07772). This investigation adhered to the guidelines of the International Conference on Harmonization‐Good Clinical Practice [ICH‐GCP: E6(R2)] and aligned with the principles outlined in the Declaration of Helsinki. Data collection consisted of the age at Fontan operation, duration of Fontan operation, cardiac diagnosis, previous Glenn operation and type of Fontan operation. Laboratory tests included complete blood count (CBC), renal function (BUN, Cr), liver function test (LFT), gamma‐glutamyl transpeptidase (GGT), alpha‐fetoprotein (AFP) and lipid profile. In addition, a mitochondrial function test was investigated using isolated PBMCs. A cardiovascular evaluation was performed including echocardiography and cardiac catheterization. The METAVIR scoring system by liver biopsy was used to assess the severity of fibrosis in liver tissue (Figure 1), including F0—no fibrosis; F1—portal fibrosis; F2—bridging fibrosis with few septa; F3—bridging fibrosis with many septa; and F4—cirrhosis. The Fontan patients in this study were classified with the METAVIR score as follows; F0/F1—no/mild fibrosis; F2—moderate fibrosis; and F3/F4—cirrhosis.

### 2.2 Isolation of peripheral blood mononuclear cells (PBMCs)

The EDTA‐coated tube was used to collect blood, and a Ficoll density gradient centrifugation was used to separate the PBMCs.^14^ Peripheral Blood Mononuclear Cells were used to measure cell death and mitochondrial function including mitochondrial respiration, mitochondrial oxidative stress and mitochondrial mass. The complete blood sample was centrifuged at 1000 *g* for 10 min in order to perform the Ficoll density gradient procedure, and the plasma was discarded. Red blood cells and the buffy coat were re‐dissolved in a phosphate buffer saline (PBS) solution (1‐part cells: 3‐parts PBS). The blood was then centrifuged at 400 *g* for 30 minutes after being over‐layered over a Histopaque reagent (Sigma‐Aldrich). After centrifugation, the PBMC ring at the Ficoll/plasma interface was collected and washed twice with PBS solution. The pallet was submitted for cell counting using a haemocytometer technique following the final centrifugation at 1000 *g* for 10 min.^14^

### 2.3 Cellular oxidative stress, and ratio of mitochondrial reactive oxygen species (ROS)/mitochondrial mass levels in PBMCs

Peripheral Blood Mononuclear Cells (2 × 10^5^ cells) were incubated with 2 μM of DCFH‐DA dye (Sigma) at room temperature for 20 min. Following that, flow cytometry (FACS Celesta, BD Biosciences) was used to assess the level of DCF. Moreover, PBMCs (2 × 10^5^ cells) were incubated with MitoSOX Red dye (Thermo Fisher), and co‐incubated with 50 nM of MitoTracker Green dye (Thermo Fisher) at 37°C for 30 min. Using flow cytometry (FACS Celesta, BD Biosciences), the fluorescence intensity of MitoSOX and MitoTracker Green was examined. The intensity of MitoSOX indicated mitochondrial ROS levels, then the intensity of MitoTracker green indicated mitochondrial mass levels, and finally, the ratio of MitoSOX/MitoTracker green was calculated.^15^

### 2.4 Mitochondrial oxygen consumption determination

Peripheral Blood Mononuclear Cells (2 × 10^5^ cells) were loaded into a XFe96‐well plate and centrifuged at 200 *g* for 1 min. Then, the base medium buffer supplemented with 2 mM of l‐glutamine was added. The PBMCs were incubated for 30 min at 37°C before starting the assay. Mitochondrial function was assessed using a mitochondrial stress test kit (Agilent), and the oxygen consumption rate (OCR) was evaluated using a high‐throughput automated 96‐well extracellular flux analyzer (XFe96; Agilent). The sequential reagents were added as follows: 1 μM‐Oligomycin was first added, followed by 2 μM‐FCCP, and finally 0.5 μM‐Rotenone/antimycin A. Automatic assessments of basal respiration, ATP production, maximal respiration, and spare respiratory capacity were made by the analytical software (Wave; Agilent Seahorse).^15^

### 2.5 Cell death determination in PBMCs

FITC‐AnnexinV/PE‐PI dye (BD Bioscience) was used to stain PBMCs (2 × 105 cells), and they were incubated for 15 min. Then, the cells were analysed using flow cytometry. The percentage of live cells, apoptosis (FITC‐stained cells), necroptosis (FITC and PE‐stained cells) and necrosis (PE‐stained cells) were analysed.^16^

### 2.6 Serum biomarkers for liver fibrosis

The serum biomarkers for liver fibrosis consisted of the aspartate amino trans‐ferase‐to‐platelet ratio index (APRI) score, fibrosis‐4 (FIB‐4) score and Forns Index. The APRI score was calculated using aspartate aminotransferase (AST), and platelet count.^17^ The FIB‐4 score used age, AST, alanine aminotransferase (ALT), and platelet count.^18^ Forns Index was determined using age, platelet count, GGT and cholesterol.^19^

### 2.7 Liver ultrasound and elastography

Radiologist performed an ultrasound on the upper abdomen. Low‐frequency curved‐array transducers C1‐6 (2–5 MHz) were utilized in the GE‐E 10 ultrasound system. The patients fasted for at least 6 h before the upper abdomen ultrasound. A heterogenous parenchymal echotexture was used to define chronic parenchymal liver disease; splenomegaly or ascites were used to diagnose portal hypertension; surface nodules, blunt margins, right hepatic lobe atrophy, caudate or left hepatic lobe hypertrophy, and liver nodules were used to diagnose cirrhosis.

A radiologist used ultrasound liver elastography to assess the stiffness of the liver. Low‐frequency curved‐array transducers C1‐6 (2–5 MHz) were utilized in the GE‐E 10 ultrasound system. The patients fasted for at least six hours prior to ultrasound liver elastography. The patients underwent B‐mode and colour Doppler ultrasound followed by a 2D shear wave. Patients were measured for liver stiffness while they were laying in the left decubitus posture with the right arm extended. The probe was inserted in the right lobe of the liver through the intercostal spaces. The chosen area of measurement was correlated with the abnormal finding on the ultrasound monitor. A value using kilopascals (kPa) was recorded and classified according to the METAVIR scoring system including F0 = no fibrosis; F1 = portal fibrosis; F2 = bridging fibrosis with few septa; F3 = bridging fibrosis with many septa; F4 = cirrhosis. The grading of severities consisted of 5.48–8.29, 8.29–9.40, 9.40–11.90, and >11.90 kPa, representing mild, moderate, and severe fibrosis and cirrhosis respectively.

### 2.8 Computerized tomography (CT) scan of the liver

On a SIEMENS Healthiness‐SOMATOM Force, an upper abdominal CT scan was carried out on Fontan patients following a defined procedure. Prior to the CT scan, the patients fasted for at least 6 h. Non‐contrast, arterial (25–30 s), post‐venous (60 s) and delay phases made up the four scan phases. Every patient received Omnipaque‐350 at a rate of 2 mL/kg in 3 mL/s. In this investigation, no oral contrast was employed.

### 2.9 Echocardiography

A cardiologist performed echocardiography to evaluate the morphology of systemic ventricle, the severity of atrioventricular valve regurgitation, systolic and diastolic function, Fontan circuit and fenestration.

### 2.10 Cardiac catheterization

A paediatric cardiologist performed cardiac catheterizations. The patients fasted for eight hours before the catheterization. Anesthesiologists were in charge of administering the sedative during catheterization. The hemodynamic measurements were recorded, including mean pulmonary arterial pressure, Fontan pressure, hepatic vein pressure, systemic ventricular end‐diastolic pressure and transpulmonary gradient pressure. Moreover, the evaluated findings consisted of a clot in Fontan circuit/pulmonary artery/cardiac chamber, pulmonary artery size, fenestration and venovenous collateral vessels.

### 2.11 Liver biopsy

An interventional radiologist performed a percutaneous liver biopsy under ultrasound supervision. (Philip, the Netherlands). The appropriate pre‐operative laboratory tests were a platelet count of more than 50,000 cells/mm^3^ and an INR of less than 1.5 according to the Society of Interventional Radiology consensus guidelines. In every patient, warfarin and aspirin were temporally discontinued for 5 days before liver biopsy. The liver biopsy was done at the right lobe of the liver, using an intercostal approach. Parenchymal liver tissue was obtained from every patient and the nodule tissue biopsy was additionally performed in some patients with liver nodule(s) appearing on the ultrasound of the upper abdomen. Two pieces of adequate tissue (each 2 × 0.1 cm^2^) were collected via Bard^@^ Mission, disposable core biopsy, Semi‐automatic needle, and needle gauge 18G × 16 cm. Formalin‐fixed, paraffin‐embedded (FFPE) tissue was stained with routine haematoxylin and eosin for histopathologic evaluation and Masson's trichrome for fibrosis interpretation. They were evaluated by the pathologist. The METAVIR fibrosis system was applied for fibrosis staging as follows: F0 = no fibrosis; F1 = portal fibrosis; F2 = bridging fibrosis with few septae; F3 = bridging fibrosis with many septae; F4 = cirrhosis.

### 2.12 Statistical analysis

Continuous data were presented as mean ± standard deviation in normally distributed data, and for non‐normally distributed data, the median (25th to 75th interquartile range, IQR) was used. Numbers (percentages) were used to represent categorical data. The differences in continuous data between groups were compared using one‐way anova or Kruskal–Wallis test as appropriate. Categorical data were compared using the chi‐squared test. Multivariable ordinal logistic regression was used for the factors associated with the severity of liver fibrosis. Statistical significance was defined as a *p*‐value of <0.05. All statistical analysis was performed using Stata (StataCorp).

## 3 RESULTS

### 3.1 Demographic parameters

Thirty‐seven patients (18 male) received cardiac and hepatic evaluations post‐Fontan operation in this study. The median age was 19 years (IQR 16–22), and the median body weight was 50 kg (IQR 45–54.5). The median age at Fontan operation was 6 years (IQR 5.5–8.4) and the median time elapsed since Fontan's operation was 12 years (IQR 9.6–14.8). The median oxygen saturation at the time of recruitment was 94% (IQR 92–95). Almost all patients (90%) did not develop any symptoms. The diagnoses were as follows: 14 cases (38%) with tricuspid atresia; six cases (17%) with double inlet left ventricle; four cases (11%) with mitral atresia; two cases (5%) with unbalanced atrioventricular septal defect; two cases (5%) with heterotaxy syndrome; two cases (5%) with hypoplastic right ventricle and seven cases (19%) with other diagnoses. A bidirectional Glenn shunt before total cavopulmonary connection was performed in 28 patients (76%), consisting of pulsatile unilateral (eight cases), non‐pulsatile unilateral (17 cases), pulsatile bilateral (one case) and non‐pulsatile bilateral (two cases). Most types of Fontan operation were extracardiac conduit (35 cases; 94.6%) and the majority size of polytetrafluoroethylene (PTFE) for conduit was 20 mm (25 cases; 69%). Twenty‐three patients (64%) had undergone a fenestration and the predominant size of these fenestrations was 6 mm. (12 cases; 36%). The baseline characteristics did not significantly differ among the groups, and demographic data are shown in Table 1.

**TABLE 1:** Baseline characteristics between no/mild fibrosis, moderate fibrosis and cirrhosis.

Characteristics	No/mild fibrosis group (*n* = 7)	Moderate fibrosis group (*n* = 7)	Cirrhotic group (*n* = 23)	*p*‐Value
Male, *n* (%)	3 (42.8)	4 (57.1)	11 (47.8)	0.86
Age (years), median (IQR)	16.6 (15.4–28.6)	18 (15.9–19.1)	20.1 (17.1–24.6)	0.21
Age at Fontan (years), median (IQR)	6.3 (5.9–12.3)	6.7 (5.7–8.0)	6.2 (5.3–9.1)	0.76
Time elapse since Fontan (years), median (IQR)	10.1 (9.1–18.5)	9.9 (8.6–13.7)	13.2 (10.8–14.9)	0.38
Weight (kgs), median (IQR)	47.9 (40–50.2)	50.5 (48.9–69.5)	51 (44.9–55)	0.22
Oxygen saturation (%)	94 (86–96)	93 (89–95)	94 (92–95)	0.77
Diagnosis, *n* (%)				
Tricuspid atresia	2 (28.6)	1 (14.3)	11 (47.8)	0.06
Hypoplastic RV	0	2 (28.6)	0	
DILV	1 (14.2)	1 (14.3)	4 (17.4)	
Mitral atresia	2 (28.6)	0	2 (8.7)	
Heterotaxy syndrome	0	2 (28.6)	0	
Unbalanced AVSD	0	0	2 (8.7)	
Other	2 (28.6)	1 (14.3)	4 (17.4)	
Previous Glenn shunt, *n* (%) 0.85				
Pulsatile				
Unilateral	1 (20)	3 (42.9)	4 (25)	
Bilateral	0	0	1 (6.3)	
Non‐pulsatile				
Unilateral	3 (60)	4 (57.1)	10 (62.4)	
Bilateral	1 (20)	0	1 (6.3)	
Type of Fontan operation, *n* (%)				
Atriopulmonary connection	0	0	1 (4.3)	0.62
Lateral tunnel	1 (14.3)	0	0	
Extracardiac conduit	6 (85.7)	7 (100)	22 (95.7)	
Fenestration, *n* (%)	4 (57.1)	7 (100)	12 (54.6)	0.09
Size of fenestration, No., *n* (%)				
4	1 (14.3)	0	2 (9.5)	0.33
5	0	0	2 (9.5)	
6	2 (28.6)	4 (80)	6 (28.6)	
8	1 (14.3)	1 (20)	2 (9.5)	
9	1 (14.3)	0	0	
Cardiopulmonary bypass time, min	103.5 (85.5–168.8)	87 (67.8–161.3)	111 (87–146)	0.55
Aortic cross‐clamp time, min	46 (20.3–72.8)	31.5 (14.3–45)	46 (0–60)	0.58

Abbreviations: AVSD, atrioventricular septal defect; DILV, double inlet left ventricle; min, minute; RV, right ventricle.

All patients received percutaneous liver biopsies. Based on the METAVIR scoring system by liver histology, the patients were divided into three groups including no or mild liver fibrosis (F0/F1, *n* = 7), moderate liver fibrosis (F2, *n* = 7) and severe fibrosis or cirrhosis (F3/F4, *n* = 23).

## 4 MODALITIES FOR ASSESSMENT OF THE SEVERITY OF LIVER FIBROSIS

### 4.1 Laboratory tests

Liver and renal function tests did not significantly differ among groups (Table 2). The cirrhotic group had significantly decreased haemoglobin and haematocrit levels when compared with both groups (*p* = 0.01 and 0.005).

**TABLE 2:** Laboratory tests between no/mild fibrosis, moderate fibrosis and cirrhosis.

Characteristics	No/mild fibrosis group (*n* = 7)	Moderate fibrosis group (*n* = 7)	Cirrhotic group (*n* = 23)	*p*‐Value
Haemoglobin (g/dL)	15.7 (14.9–16.6)	15.9 (15.6–17.8)*	14.4 (13.7–15.6)*	**0.01**
Haematocrit (%)	46.2 (45.2–50.3)	48.7 (47.8–54.9)*	43.7 (41.9–45.5)*	**0.005**
Platelet × 10^9^/L	181,000 (168,000–196,000)	250,000 (177,000–299,000)	212,000 (177,000–241,000)	0.26
Creatinine	0.7 (0.6–0.8)	0.9 (0.7–1.1)	0.7 (0.7–0.9)	0.33
Albumin (g/dL)	4.6 (4.5–5)	4.5 (4.4–4.8)	4.6 (4.3–4.9)	0.93
AST (U/L)	21 (20–26)	26 (18–31)	24 (20–29)	0.61
ALT (U/L)	17 (15–20)	19 (14–30)	23 (17–27)	0.15
ALP	101 (61–235)	139 (92–161)	120 (83–149)	0.88
Total bilirubin (μmol/L)	1.0 (0.7–1.1)	0.9 (0.7–1.9)	0.8 (0.6–1.4)	0.91
Direct bilirubin (μmol/L)	0.4 (0.3–0.5)	0.4 (0.3–0.8)	0.4 (0.3–0.5)	0.95
GGT (U/L)	56 (36–78)	71 (66–114)	78 (38–169)	0.42
APRI score	0.29 (0.25–0.34)	0.26 (0.21–0.39)	0.29 (0.23–0.40)	0.84
FIB‐4 score	0.54 (0.45–0.67)	0.45 (0.34–0.60)	0.47 (0.36–0.78)	0.56
Forns index	2.62 (2.13–3.47)	1.70 (1.04–3.54)	2.93 (1.83–4.68)	0.23

Abbreviations: ALP, alkaline phosphatase; ALT, alanine aminotransferase; APRI, aspartate amino transferase‐to‐platelet ratio index; AST, aspartate aminotransferase; FIB‐4, fibrosis‐4; GGT, gamma‐glutamyl transpeptidase. *p < 0.05 for moderate fibrosis group versus cirrhotic group.

### 4.2 Serum biomarkers for liver fibrosis

The serum biomarkers for liver fibrosis in this study consisted of the APRI score, FIB‐4 score and Forns Index, and these scores showed no significant difference among groups (*p* = 0.84 for APRI score, *p* = 0.56 for FIB‐4 score, and *p* = 0.23 for Forns Index) (Table 2).

### 4.3 Liver ultrasound and elastography

The majority of liver ultrasounds in the no‐significant fibrosis group was heterogenous aspect parenchyma (57%). Cirrhosis imaging was identified the most in the cirrhotic group (53%).

For ultrasound liver elastography, we demonstrated that the value of kPa did not significantly differ among groups (*p* = 0.92) (Table 3), and the highest level of elasticity was found in the no‐significant fibrosis group.

**TABLE 3:** Parameters of imaging for evaluate FALD between no/mild fibrosis, moderate fibrosis and cirrhosis.

Characteristics	No/mild fibrosis group (*n* = 7)	Moderate fibrosis group (*n* = 7)	Cirrhotic group (*n* = 23)	*p*‐Value
Ultrasound upper of abdomen, *n* (%)				
Heterogenous aspect parenchyma	4 (57.1)	3 (42.9)	7 (36.8)	0.72
Cirrhosis	1 (14.3)	3 (42.9)	10 (52.6)	0.34
Liver nodules	2 (28.6)	2 (28.6)	2 (10.5)	0.28
Ultrasound liver elastography (kPa), median (IQR)	13.8 (7.9–17.7)	11.6 (10.8–12.3)	10.3 (9.2–20.1)	0.92
Computerized tomography scan, *n* (%)				
Cirrhosis	3 (42.9)	2 (28.6)	11 (47.8)	0.74
Liver nodules	4 (57.1)	4 (57.1)	15 (65.2)	1.00

### 4.4 Computerized tomography (CT) scan of the liver

Similar results with the liver ultrasound revealed that the cirrhosis and liver nodules findings according to the CT scan of the liver did not significantly differ among groups (*p* = 0.74 and 1.0) (Table 3). The cirrhotic picture was identified more predominantly in the cirrhotic group and liver nodules were equally demonstrated in all groups.

### 4.5 Six‐minute walk

We revealed that the six‐minute walk (meters) was not significantly different among groups (*p* = 0.12) (Table 4).

**TABLE 4:** Hemodynamic study after Fontan operation between no/mild fibrosis, moderate fibrosis and cirrhosis.

Characteristics	No/mild fibrosis group (*n* = 7)	Moderate fibrosis group (*n* = 7)	Cirrhotic group (*n* = 23)	*p*‐Value
6‐min walk, meters	360 (300–420)	420 (380–504)	426 (390–450)	0.12
Echocardiography				
Single ventricular function, *n* (%)				
Poor	2 (28.6)	1 (14.3)	7 (30.4)	0.63
Fair	2 (28.6)	1 (14.3)	8 (34.8)	
Normal	3 (42.9)	5 (71.4)	8 (34.8)	
AV valve regurgitation, *n* (%)				
No	4 (57.1)	4 (57.1)	10 (45.5)	1.00
Mild	2 (28.6)	2 (28.6)	8 (36.4)	
Moderate	1 (14.3)	1 (14.3)	4 (18.2)	
Cardiac catheterization, mmHg, mean (SD)				
Hepatic vein	14 (12.8–15.3)	14 (12–19)	15 (14–17)	0.53
IVC	12.5 (11–15)	13 (12–17)	15 (13–17)	0.31
Fontan circuit	13.5 (11–15.8)	13 (11–17)	15 (13–17)	0.67
Pulmonary artery	14 (12–15.5)	13 (11–17)	15 (13–17)	0.79
SVEDP	11 (7.3–15)	10 (8–12)	10 (9–12)	0.74
PVRi	1.2 (0.5–3.2)	2.3 (0.6–2.6)	1.6 (1.0–2.3)	0.80
Transpulmonary gradients	2 (0.75–7)	5 (2–6)	4 (3–6)	0.64

Abbreviations: AV, atrioventricular; IVC, inferior vena cava; PVRi, pulmonary vascular resistance index; SVEDP, single ventricular end‐diastolic pressure.

### 4.6 Echocardiography

For echocardiographic findings after the Fontan operation, the cirrhotic group had the most single ventricle dysfunction (fair and poor function; 65%) when compared with the other two groups (*p* = 0.63) (Table 4). The majority of patients in the no‐significant fibrosis group had no atrioventricular valve regurgitation.

### 4.7 Cardiac catheterization

In this study, the patients received a cardiac catheterization along with other modalities. The parameters consisting of hepatic venous pressure, inferior vena cava (IVC) pressure, Fontan pressure, mean PAP, SVEDP, PVRi, NAKATA index, transpulmonary gradient pressure, were not significantly different among groups (Table 4).

### 4.8 Mitochondrial function in PBMCs

**FIGURE 2:**
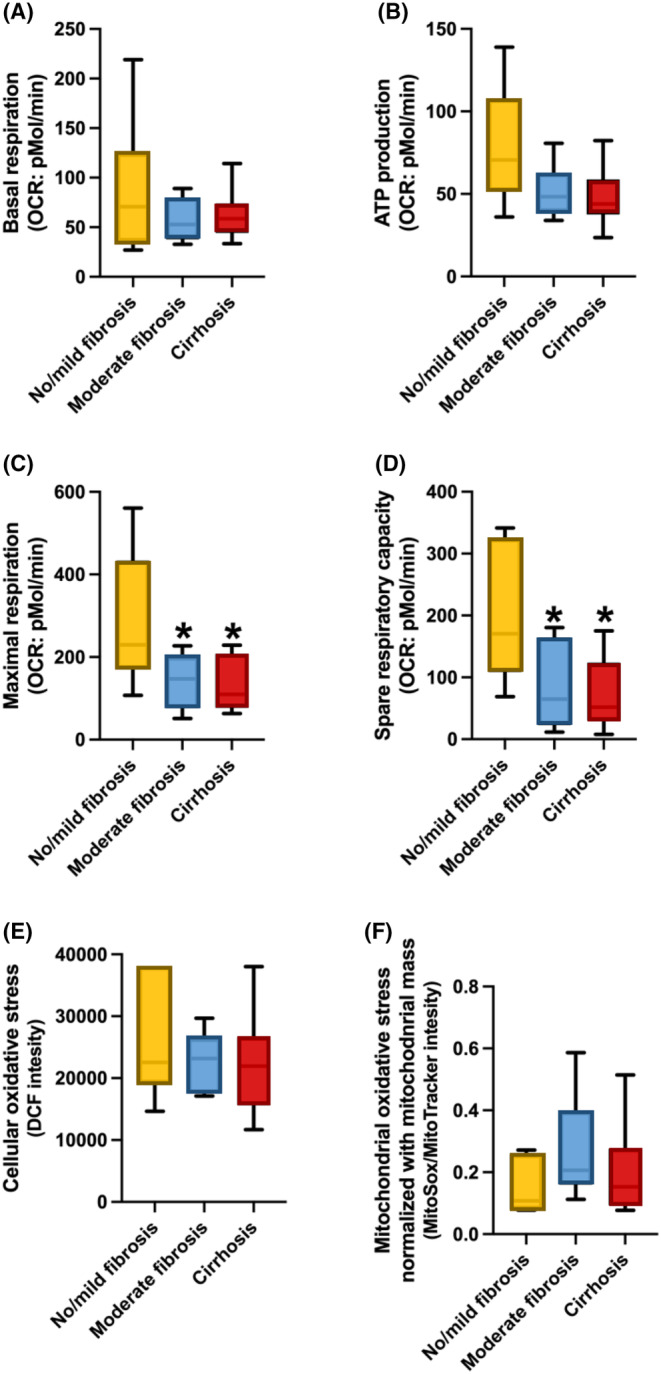
Mitochondrial function in PBMCs from Fontan patients with No/Mild fibrosis, moderate fibrosis, and cirrhosis. *p < 0.05 versus No/Mild fibrosis in Maximal respiration and Spare respiratory capacity. One‐way anova test was used.

Data from mitochondrial respiration analysis in PBMCs showed that basal respiration and ATP production were comparable among groups (Figure 2A,B). The study revealed that the F3/F4 and F2 group exhibited notably decreased levels of maximal respiration and spare respiratory capacity compared to the F0/F1 group. (*p* = 0.005, 0.03 and 0.004, 0.03, respectively) (Figure 2C,D).

**FIGURE 3:**
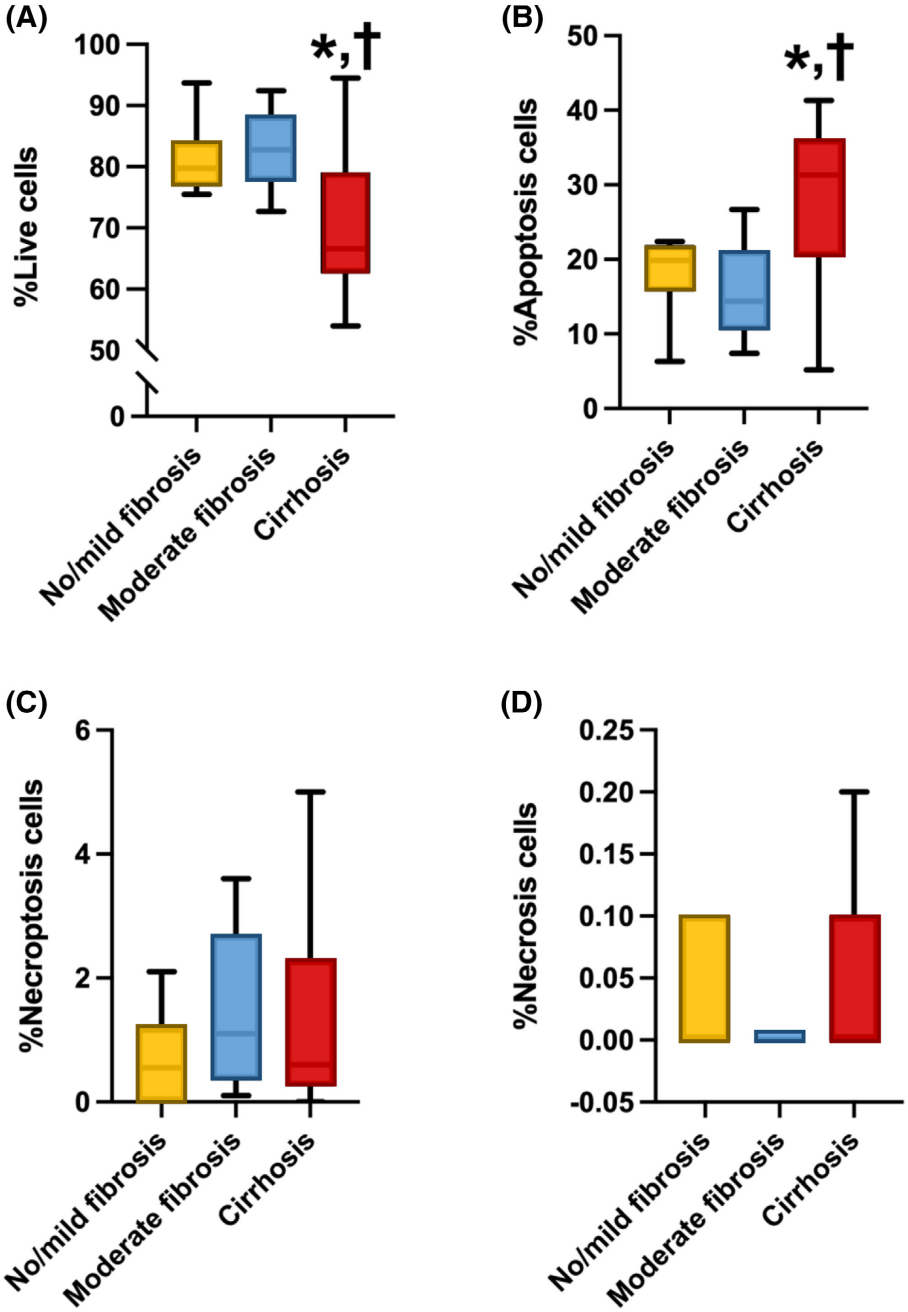
Proportion of cell death patterns in PBMCs from Fontan patients with No/Mild fibrosis, moderate fibrosis, and cirrhosis. *p < 0.05 versus No/Mild fibrosis. ^†^ *p* < 0.05 versus moderate fibrosis. Both in % Live cells and % Apoptosis cells. One‐way anova test was used.

For oxidative stress parameters, our results demonstrated that cellular and ratio of mitochondrial ROS/mitochondrial mass were not significantly different among groups (Figure 2E,F). Moreover, our study demonstrated a significant decrease in the percentage of live cells within the F3/F4 group when compared to both the F2 and F0/F1 groups (*p* = 0.03 and 0.04, respectively). Additionally, the F3/F4 group exhibited a significantly higher percentage of apoptosis, compared with the F2 and F0/F1 groups (*p* = 0.01 and 0.05, respectively) (Figure 3A,B). No significant differences were observed among the groups in terms of the percentages of necroptosis and necrosis (Figure 3C,D). The results of a multivariable ordinal logistic regression analysis revealed a significant association between the severity of liver fibrosis and several factors. Specifically, maximal respiration, spare respiratory capacity, live cells and apoptosis demonstrated a statistically significant relationship with liver fibrosis severity (*p* = 0.013, 0.007, 0.009, and 0.008, respectively) (Table 5).

**TABLE 5:** Multivariable ordinal logistic regression for factors associated with the severity of liver fibrosis.

Factors*	Multivariate	
	Adjusted odd ratio (95% CI)	*p*‐Value
Maximal respiration	0.9 [0.97–0.99]	0.013
Spare respiratory capacity	0.9 [0.97–0.99]	0.007
Live cells	0.8 [0.82–0.97]	0.009
Apoptosis	1.1 [1.03–1.23]	0.008

* Adjusted with age and gender.

**FIGURE 4:**
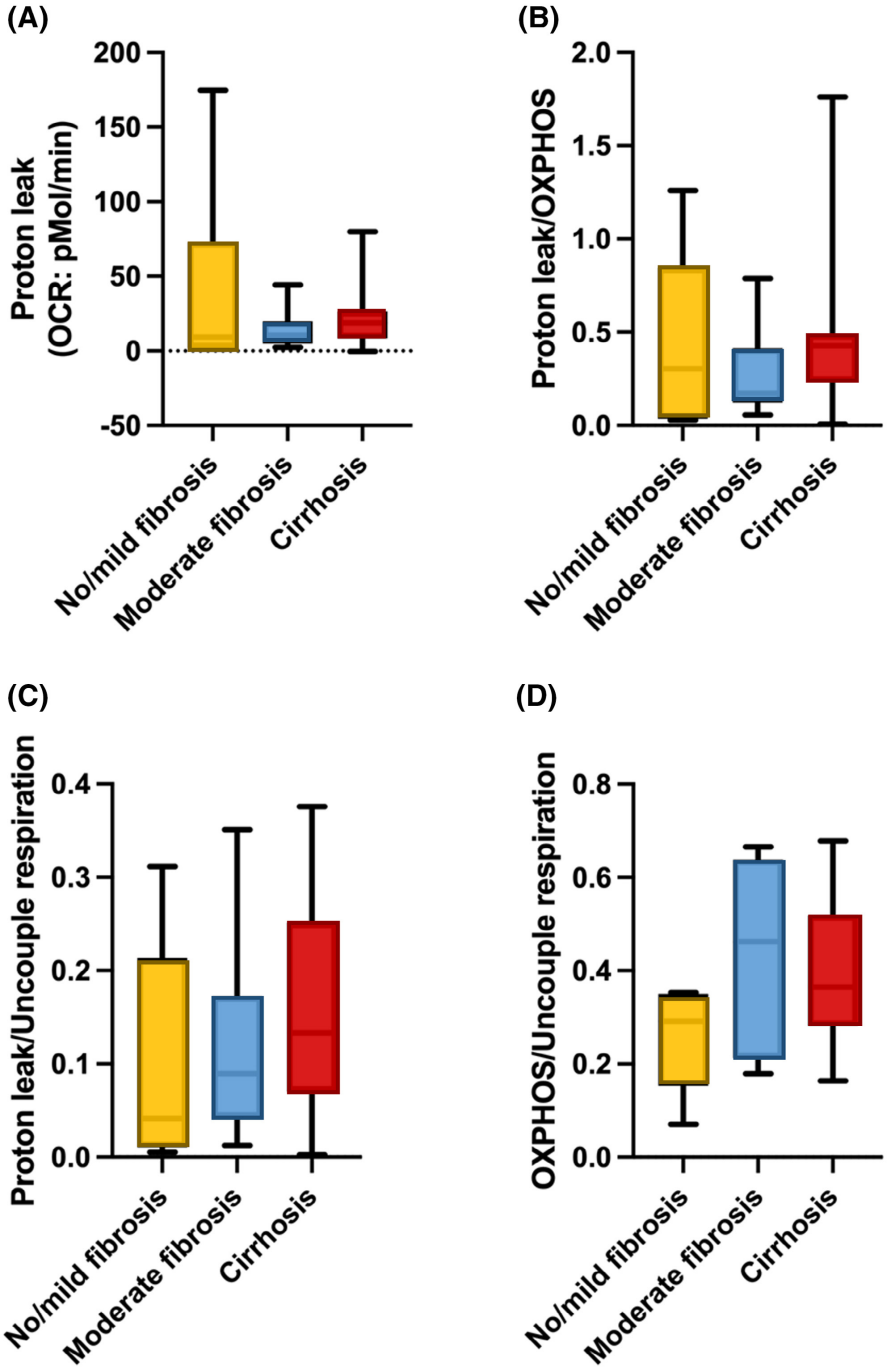
The analysis of proton leak in PBMCs in different stages of liver fibrosis in patients after the Fontan operation. (A) Proton leak, (B) ratio of proton leak/oxidative phosphorylation respiration, (C) ratio of proton leak/uncoupled synthesis, (D) oxidative phosphorylation/uncoupled respiration. OXPHOS, oxidative phosphorylation.

In addition, we observed no notable distinctions in Proton leak, Proton leak/Oxidative phosphorylation respiration, Proton leak/Uncoupled synthesis and Oxidative phosphorylation/Uncoupled respiration across the three groups of liver fibrosis in Fontan patients (Figure 4A–D).

## 5 DISCUSSION

In Fontan patients, the findings of this study indicate that mitochondrial respiration, specifically maximal respiration and spare respiratory capacity, exhibited a significant decrease in the moderate (F2) and cirrhosis (F3/F4) groups, compared with the group with no significant fibrosis (F0/F1). Additionally, the cirrhosis group showed increased levels of apoptosis and decreased levels of live cells, indicating heightened oxidative stress, compared to both the moderate and no significant fibrosis groups. Since the degree of liver fibrosis was linked to mitochondrial dysfunction and oxidative stress in PBMCs in Fontan patients, it could potentially be the novel biomarker to screen for early‐stage liver fibrosis in these patients.

Several studies reported a correlation between abnormal serum biomarkers and the stages of FALD. The most common blood test abnormality, which many studies recognized as an earlier change despite other blood tests being normal, was elevated gamma‐glutamyl transpeptidase (GGT).^20^, ^21^, ^22^, ^23^, ^24^, ^25^, ^26^, ^27^ Gamma‐glutamyl transpeptidase is the marker representing cholestasis. Therefore, elevated GGT could be described as liver congestion in the early stage of FALD. In this study, the GGT level was greater in the moderate fibrosis and cirrhotic groups than in the no‐significant fibrosis group, but not by a statistically significant amount. In addition, our study found that the cirrhotic group had significantly decreased haemoglobin and haematocrit levels, when compared with both groups. No study has yet reported this finding. For the serum biomarker for liver fibrosis, numerous studies reported that liver fibrosis showed a correlation with an increase in APRI score,^28^, ^29^, ^30^, ^31^, ^32^ FIB‐4 score,^20^, ^33^ and Forns Index.^32^, ^34^ Unfortunately, these serum biomarkers were not significantly different among the groups in this study. It could be explained that the serum biomarkers of liver fibrosis due to hepatitis would not be applicable for use with a unique hemodynamic post‐Fontan operation where the main causes were congestive hepatopathy along with liver ischemia.

The ultrasound of the upper abdomen was the non‐invasive modality that was used to evaluate the liver parenchyma, a sign of portal hypertension, and cirrhosis. The most common findings in Fontan patients were heterogenous liver parenchyma.^23^, ^24^, ^27^, ^35^ Some studies found that these patients also had indicators of portal hypertension, and cirrhosis.^31^, ^36^, ^37^ This study reported all ultrasound findings among participants. However, no significant difference was found among groups in our study. It could be postulated that this modality was good at screening Fontan patients but lacked delineated details for classifying the severity of liver fibrosis. Furthermore, it was limited to obese patients. In addition, ultrasound liver elastography was the new modality for evaluating liver stiffness. We demonstrated that ultrasound liver elastography (kPa) could not significantly differentiate the severity of fibrosis. This modality could not discern between liver congestion, liver fibrosis, and cirrhosis.

The upper abdominal CT scan or MRI was another non‐invasive modality that could demonstrate more details and decrease the subjective interpretation of each radiologist's opinion compared to the ultrasound. These modalities revealed the findings including liver parenchymal enhancement,^26^, ^27^ portal hypertension,^28^, ^30^ cirrhosis,^37^, ^38^ and liver nodules.^39^, ^40^ Fontan patients in this study reported all findings. However, these modalities could not differentiate the severity of liver fibrosis. These modalities were appropriate for describing the detail of portal hypertension and liver nodules, but weak in classifying the severity of liver fibrosis.

Cardiac catheterization is an important modality used to reliably evaluate the hemodynamic status of the post‐Fontan operation. Many studies demonstrated elevated central venous pressure^22^, ^40^, ^41^ and low cardiac output state,^37^, ^42^ supporting the hemodynamic theory in Fontan patients. As evidenced by the fact that hepatic venous pressure exceeded the normal hepatic pressure of 5 mmHg, our study demonstrated that all Fontan patients acquired hepatic venous hypertension.^43^ This could be explained by heart failure due to continually repeated liver injury, leading to liver change as time elapsed since the Fontan operation. Unfortunately, the hemodynamic recording from cardiac catheterization did not significantly differ among the severities of liver fibrosis groups.

Mitochondrial diseases are a group of diseases which indicated defects in oxidative phosphorylation such as respiratory failure, cardiomyopathy, liver failure, renal failure, and diabetes mellitus.^44^ The relationship between mitochondrial function and cardiac dysfunction during chronic stress or heart failure is called sterile inflammation.^45^ After the Fontan operation, all patients developed sudden heart failure and the hepatic sinusoid insult by the elevated pressure from central venous hypertension which was a unique hemodynamic pattern. Thus, in this study, we examined the mitochondrial function and oxidative stress in PBMCs with the severity of liver fibrosis by liver biopsy. Our findings demonstrated that moderate (F2) and cirrhosis (F3/F4) groups exhibited a significant decrease in mitochondrial respiration, including maximal respiration and spare respiratory capacity, in comparison to the group with no significant fibrosis (F0/F1). Furthermore, the cirrhosis group demonstrated elevated apoptosis levels and reduced live cell levels, suggesting increased oxidative stress when compared to both the moderate and no significant fibrosis groups. Indeed, these findings suggest a potential association between changes in mitochondrial function and oxidative stress in PBMCs and the severity of liver fibrosis in Fontan patients. The observed significant decrease in mitochondrial respiration parameters, such as maximal respiration and spare respiratory capacity, in patients with moderate and advanced liver fibrosis (cirrhosis) implies that mitochondrial dysfunction may play a role in the development and progression of liver fibrosis in Fontan patients. This therefore highlights the importance of further investigation in the mechanisms underlying mitochondrial alterations and their impact on liver health in this patient population.

### 5.1 Limitations and gaps in current knowledge and future direction for liver fibrosis screening in Fontan patients

The limitation of our study pertains to the measurement of mitochondrial respiration, which was conducted in PBMCs and represents a global assessment of mitochondrial function. Therefore, mitochondrial respiration in liver tissue using a Clark‐type electrode is suggested in a future study for a more comprehensive evaluation. However, this study is the first to identify the novel serum biomarker for early liver fibrosis screening in Fontan patients compared to the METAVIR scoring system by liver biopsy. It could pave the way to understanding the progression of FALD stages beginning with the early post‐Fontan operation. Moreover, this biomarker might be used for differentiating the early stages of FALD from higher stages, and that it could be an important biomarker for clinicians' decision‐making process for appropriate management in the future. Further prospective study is warranted to support the results of these findings and their use in a clinical setting.

## 6 CONCLUSION

The severity of liver fibrosis in Fontan patients is strongly linked with mitochondrial dysfunction in PBMCs. Mitochondrial activity and apoptosis could be possible to use as a novel marker to detect and monitor the progression of liver fibrosis in those patients.

## AUTHOR CONTRIBUTIONS

**Saviga Sethasathien:** Conceptualization (lead); data curation (lead); formal analysis (lead); methodology (lead); writing – original draft (lead); writing – review and editing (lead). **Krit Leemasawat:** Conceptualization (supporting); methodology (equal); supervision (supporting); writing – review and editing (equal). **Suchaya Silvilairat:** Conceptualization (equal); formal analysis (supporting); methodology (lead); visualization (lead). **Rekwan Sittiwangkul:** Conceptualization (equal); methodology (supporting); supervision (supporting). **Krit Makonkawkeyoon:** Conceptualization (supporting); supervision (supporting). **Apinya Leerapun:** Conceptualization (equal); supervision (supporting). **Sarawut Kongkarnka:** Conceptualization (equal); formal analysis (equal); methodology (lead); supervision (equal). **Nakarin Inmutto:** Conceptualization (equal); formal analysis (equal); methodology (lead); supervision (equal). **Supanai Suksai:** Methodology (equal). **Nattayaporn Apaijai:** Formal analysis (equal); methodology (lead); writing – review and editing (supporting). **Siriporn C. Chattipakorn:** Conceptualization (equal); formal analysis (equal); funding acquisition (supporting); methodology (equal); supervision (supporting); writing – review and editing (supporting). **Nipon Chattipakorn:** Conceptualization (equal); formal analysis (equal); funding acquisition (lead); methodology (equal); supervision (lead); visualization (equal); writing – review and editing (lead).

## FUNDING INFORMATION

This work was supported by the Research Chair Grant from the National Science and Technology Development Agency Thailand (to NC), the Distinguished Research Professor Grant from the National Research Council of Thailand (N42A660301 to SCC), the Chiang Mai University Center of Excellent Award (to NC), and the Thailand Science Research and Innovation‐Chiang Mai University (Fundamental Fund 2565 to KL and SS).

## CONFLICT OF INTEREST STATEMENT

The authors declare no conflicts of interest.

## Data Availability

Research data will be available upon reasonable request. All requests should be submitted to the corresponding author who will review them with the other investigators for consideration. A data use agreement will be required before the release of participant data and Institutional Review Board approval as appropriate.
